# *Mycoplasma synoviae* LP78 is a fibronectin/plasminogen binding protein, putative adhesion, and potential diagnostic antigen

**DOI:** 10.3389/fmicb.2023.1335658

**Published:** 2024-01-09

**Authors:** Shuizhong Han, Ying Wang, Lizhen Wang, Wenchi Chang, Bo Wen, Junyang Fang, Xiaolan Hou, Xuefeng Qi, Jingyu Wang

**Affiliations:** ^1^College of Veterinary Medicine, Northwest A&F University, Yangling, China; ^2^College of Food and Drugs, Luoyang Polytechnic, Luoyang, China

**Keywords:** *Mycoplasma synoviae*, LP78, adhesion, fibronectin, plasminogen, ELISA, antibody

## Abstract

*Mycoplasma synoviae* (*M. synoviae*) is one of the major poultry pathogens causing infectious synovitis, airsacculitis, a high incidence of shell breakage, and egg production loss. However, the pathogenesis of *M. synoviae* remains unclear. Adhesion of mycoplasmas to host cells is a crucial step in infection and colonization. The purpose of this study was to determine the adhesive function of a putative P80 family lipoprotein (LP78) and evaluate its application in the detection of antibodies against *M. synoviae*. Recombinant LP78 (rLP78) was expressed in the supernatant component of *Escherichia coli* and mouse anti-rLP78 serum was prepared. Bioinformatic analysis and western blotting results revealed that LP78 was conservative among *M. synoviae* strains. It was distributed not only in the cytoplasm but also on the membrane of *M. synoviae* through western blotting and indirect immunofluorescence (IFA). The adherence of *M. synoviae* to DF-1 cells was significantly inhibited by mouse anti-rLP78 serum (*p* < 0.01). IFA revealed that rLP78 adhered to DF-1 cells, and this adherence was prevented by mouse anti-rLP78 serum. Furthermore, rLP78 was found to bind to the DF-1 cells membrane proteins in a dose-dependent manner by enzyme-linked immunosorbent assay (ELISA). Screening of DF-1 cells membrane proteins by western blotting showed that proteins with molecular weight of 35–40 kDa and 55–70 kDa bound to rLP78. Moreover, rLP78 was identified to be a fibronectin/plasminogen binding protein. The sensitivity and specificity of rLP78-based iELISA were 85.7 and 94.1%, respectively. The maximum dilution of positive serum (HI titer, 1:128) detected via rLP78-based iELISA was 1:6,400, whereas that detected using a commercial ELISA kit was 1:12,800–1:25,600. Both rLP78-based iELISA and the commercial ELISA kit detected seroconversion after 7 days of challenge and immunization. No cross-reactivity with positive sera against other avian pathogens was observed in rLP78-based iELISA. Collectively, these results indicate that LP78 is a fibronectin/plasminogen-binding adhesion protein of *M. synoviae* and a potential diagnostic antigen. The present study will facilitate a better understanding of the pathogenesis of *M. synoviae* and the development of new diagnostic.

## Introduction

*Mycoplasma synoviae* (*M. synoviae*) is one of the most important poultry pathogens, leading significant economic losses to the poultry industry worldwide. *M. synoviae* infection can cause infectious synovitis, airsacculitis, a high incidence of shell breakage, and egg production loss (Kleven et al., 1975; Cisneros-Tamayo et al., 2020). Substantial variations in pathogenicity and tissue tropism are observed among *M. synoviae* isolates, and some strains cause no infection whereas others cause respiratory disease or synovitis (Lockaby et al., 1998; Lockaby and Hoerr, 1999). It is challenging to diagnose *M. synoviae* infection on-site, especially when additional respiratory agents are present (Kleven et al., 1975; Hopkins and Yoder, 1982). Furthermore, breeder hens infected with *M. synoviae* can spread the disease vertically to progeny and laterally through direct contact via the respiratory tract (Feberwee et al., 2021). Upon infection, *M. synoviae* can persistently colonize the tracheas of chickens, even throughout the production period (Olson and Kerr, 1967). In some continents, the seroprevalence of *M. synoviae* exceeds 70% in layer flocks, and the clinical and economic relevance of *M. synoviae* is superior to that of *Mycoplasma gallisepticum* (*M. gallisepticum*) (Landman, 2014; Cortes et al., 2021). However, the pathogenesis and possible virulence factors of *M. synoviae* are not yet fully known.

Adhesion of mycoplasmas to host cells is recognized as the primary requisite for infection and successful colonization (Razin and Jacobs, 1992). The loss of adhesion capacity leads to loss of infectivity, whereas reversion to cytadhering phenotype is accompanied by recovering infectivity and virulence (Krause et al., 1982, 1983). Therefore, cytadhesins play a significant role in determining the virulence of mycoplasmas. Due to the lack of cell walls, the mycoplasmas membrane components are in directed contact with the cell membranes of the host and are responsible for adherence. The non-specific initial stage of the adhesion process is determined by the microorganisms’ structure. The second stage entails the correctly binding of the microorganism-specific ligands to the relevant host cell receptors, primarily via the surface proteins (Razin and Jacobs, 1992). Variable lipoprotein hemagglutinin (vlhA), enolase, pyruvate dehydrogenase alpha (PdhA) and beta (PdhB) subunits, dihydrolipoamide dehydrogenase (PdhD), NADH oxidase and other surface proteins from *M. synoviae* have been identified to be involved in adhesion (Noormohammadi et al., 1997; Bao et al., 2014, 2021; Hu et al., 2022; Qi et al., 2022). Additionally, most microorganisms bind to a range of host extracellular matrix (ECM) components, such as fibronectin (Fn), collagen, elastin, and laminin via interactions between microbial adhesins and host cell receptors (Chagnot et al., 2012). Fn is a multifunctional glycoprotein with a high molecular weight. It can be found as an insoluble multimer in conjunction with the surfaces of eukaryotic cell, the ECM, and the basement membrane (BM), or as a soluble dimer in majority of body fluids (Henderson et al., 2011). Furthermore, bacterial can degrade tissue barriers formed by ECM and BM through fibrinolysis, which is one of the most important factors in the pathogenesis of bacterial infection. Plasminogen (Plg) is cleaved to generate active plasmin, which plays an important role in the fibrinolysis system (Bhattacharya et al., 2012). *M. synoviae* can be detected in a variety of internal organs, including trachea, lung, air sac, joint, and so on (Lockaby et al., 1998). Fn-binding proteins (FnBPs) and Plg-binding proteins (PlgBPs) are present in many species of bacteria. The inactivation or mutation of some FnBPs genes decrease the adherence or virulence (Cloward and Krause, 2009; Henderson et al., 2011; Zhao et al., 2017; Liu et al., 2023). PlgBPs will enhance the conversion of Plg to plasmin, allowing bacteria to invade more easily (Li et al., 2022; Liu et al., 2023). Until now, the binding abilities of enolase, PdhA and PdhB subunits, PdhD and NADH oxidase to Fn and Plg have been determined. In addition to the above-mentioned proteins, other proteins in *M. synoviae* may also contribute to the cytadhesion and possess Fn/Plg-binding abilities. Therefore, it is important to identify and characterize membrane-associated proteins involved in adherence and Fn/Plg-binding, since this may assist to shed light on the pathogenesis of *M. synoviae*.

Usually, adhesins are exposed on the mycoplasma cell surface and have the ability to elicit humoral responses, and then could potentially be used as a diagnostic tool. For the control and elimination of *Mycoplasma* infection, flocks should be maintained free of *Mycoplasma* infection by implementing an effective biosecurity program and a consistently applied monitoring system. Serological assays are widely used for preliminary diagnosis of *M. synoviae* infection. Until now, several serological tests, including serum plate agglutination (SPA), hemagglutination inhibition (HI), and enzyme-linked immunosorbent assay (ELISA), have been developed for monitoring flocks and detecting the outbreak of *M. synoviae* infection (Vardaman and Yoder, 1969; Thornton, 1978; Opitz et al., 1983). ELISA has been reported to have higher specificity than SPA and higher sensitivity than HI (Opitz et al., 1983). Several ELISAs based on whole cells or membrane proteins have been developed to detect antibodies against *M. synoviae* (Opitz et al., 1983; Higgins and Whithear, 1986; Opitz and Cyr, 1986). However, the cross-reactivity of these ELISAs with *M. gallisepticum* and non-specific reactions impede the development of specific serodiagnostic tests (Opitz et al., 1983; Higgins and Whithear, 1986; Opitz and Cyr, 1986). ELISA based on MSPB, the amino-terminal end of vlhA, has demonstrated good correlation with SPA without any cross-reactivity with sera against *M. gallisepticum* (Noormohammadi et al., 1999). However, a high degree of amino acid variations is observed in MSPB across strains, which affects the sensitivity of ELISA (Noormohammadi et al., 1998, 2002). Therefore, identifying novel antigens for the diagnosis of *M. synoviae* infection is necessary.

In previous study, LP78 is identified to be one of the major immunogenic proteins of *M. synoviae* and is annotated to be a P80 family lipoprotein (Bercic et al., 2008). Lipoproteins play important roles in the pathogenicity and virulence in some pathogenic mycoplasmas, and some are associated with adherence (Narat et al., 1998; Browning et al., 2011; Xiong et al., 2016). Nevertheless, few studies have been reported on the functions of LP78. We previously produced recombinant LP78 (rLP78) in *E. coli*, and the rLP78 was strongly recognized by *M. synoviae* convalescent serum. This promoted us to consider the potential roles of LP78 in cytoadherence, Fn/Plg-binding, and serological detection.

In this study, we demonstrated that LP78 is a surface located protein on *M. synoviae* and is involved in cytadhesion with Fn/Plg-binding activities. In addition, the LP78-based ELISA showed a good performance in the diagnosis of anti-*M. synoviae* antibodies. The findings of this study may facilitate a better understanding of the pathogenesis of *M. synoviae* and improve the serological diagnosis of *M. synoviae* infection.

## Materials and methods

### Bacterial strains, cell line, growth conditions, and sera

Five *M. synoviae* strains, including Strain W1, YL, HN, M6, and Q9 were isolated from chickens with severe airsacculitis or synovitis in China during 2021 to 2022. The isolates were propagated in modified Frey’s medium at 37°C with 5% CO_2_. The *E. coli* strains DH5α and BL21 (DE3) were grown in Luria–Bertani (LB) broth or on solid media. A continuous cell line of chicken embryo fibroblasts (DF-1 cells) was obtained from the American Type Culture Collection (Manassas, VA, United States) and grown in Dulbecco’s modified Eagle’s medium (DMEM; Gibco, Carlsbad, CA, United States) containing 10% fetal bovine serum (FBS; Gibco), 100 IU/mL of penicillin, and 100 μg/mL of streptomycin at 37°C with 5% CO_2_. Mouse anti-*M. synoviae* polyclonal antibody was produced in female BALB/c mice by the inoculation of a commercial inactivated *M. synoviae* vaccine (YBF-MS1 strain) (Yebio Bioengineering, co., Ltd. of Qingdao, China) via muscular routine. Convalescent sera samples were collected from commercial poultry farms with known *Mycoplasma* infection status and confirmed by a commercial *M. synoviae* antibody test kit (IDEXX, Westbrook, Maine, USA). Chicken sera against other avian pathogens, including *M. gallisepticum*, *Haemophilus paragallinarum* (*Hpg*), newcastle disease virus (NDV), avian influenza virus (AIV), infectious bronchitis virus (IBV), and infectious bursal disease virus (IBDV), were preserved in our laboratory.

### Bioinformatic analysis

The full-length sequence of the gene encoding LP78 (MSH_01690) in *M. synoviae* strain MS-H was obtained from the GenBank database (accession number: AWL84125). Molecular weight (Mw) of LP78 (MSH_01690) was computed by Expasy proteomics tool.^1^ BLASTP^2^ was used to carry out amino acid identity matches with sequences retrieved from NCBI database. SignalP-5.0 Server^3^ was conducted to predict the presence of signal peptide. Prediction of transmembrane helices was performed with TMHMM Server V. 2.0.^4^ CELLO^5^ was employed to predict protein subcellular localization. The protein sequence was submitted to the VaXiJen v2.0 server^6^ to identify antigenicity and its adhesin probability was calculated through Vaxign.^7^

### Amplification of the *lp78* gene and mutagenesis via overlap-extension PCR

The *lp78* gene contains six UGA codons that are interpreted as tryptophan codons in *M. synoviae* but as stop codons in *E. coli*. Seven primer pairs designed and synthesized by Tsingke Biotechnology Co., Ltd. (Beijing, China) were used to clone and mutate *lp78*. The primer sequences are shown in Supplementary Table S1. The genomic DNA of *M. synoviae* strain MS-H (Bioproperties Ltd., Ringwood, Victoria, Australia) was extracted using the TIANamp Bacteria DNA Kit (Tiangen, Beijing, China). Sequences that contained tryptophan codons (TGA) were subjected to site-directed mutagenesis to TGG via overlap-extension PCR. In the first PCR run, the seven primer pairs were used to amplify the genomic DNA. Seven fragments of 99 bp, 126 bp, 552 bp, 482 bp, 611 bp, 129 bp, and 398 bp in size were obtained (Supplementary Figure S1A).The amplified fragments were collected using the TIANgel Purification Kit (Tiangen, Beijing, China) and used as templates in the second PCR run. The lp78-1-F/ lp78-7-R primers were used to amplify *lp*78 gene and a fragment of 2,235 bp was obtained (Supplementary Figure S1B). The reaction mixture had a final volume of 50 μL, including 2 μL of each primer, 25 μL of 2 × PrimeSTAR Max Premix (Takara Biomedical Technology (Beijing) Co., Ltd., China), 19 μL of ultrapure water, and 2 μL of template DNA. The PCR conditions were as follows: 94°C for 10 min; 35 cycles of 95°C for 30 s, 55°C for 30 s, 72°C for 60 s, and 72°C for 10 min. The PCR product was applied to gene sequencing by Tsingke Biotechnology Co., Ltd. (Beijing, China). The mutated gene (2,235 bp) was cloned into the pET-28a (+) vector and transformed into *E. coli* DH5α and BL21 (DE3) competent cells using the heat shock method.

### Expression, purification, and identification of rLP78

*E. coli* BL21 (DE3) cells containing the recombinant plasmid were cultured in LB broth supplemented with kanamycin (50 μg/mL) at 37°C on a shaker at 200 rpm. The cells were incubated with 1-mM isopropyl β-D-1-thiogalactopyranoside (IPTG) at 22°C for 12 h. After induction, rLP78 was successfully expressed in *E.coli*, and 80-kDa protein was existed mainly in the supernatant of the bacterial lysate (Supplementary Figure S2). After centrifugation at 5,000 rpm for 10 min, the cell pellets were harvested and washed twice with tris–HCl (0.02 mol/L, pH 8.0).The cell pellets were sonicated on ice with 5-s pulses at 15-s intervals. After centrifugation, the supernatant containing the recombinant protein (rLP78) was added to an affinity chromatography column prepacked with Ni-NTA His-Bind^®^ Resin (Huiyan bio, Wuhan, China), and the recombinant protein was eluted using a linear gradient of 20–500 mM of imidazole. The recombinant protein was analyzed on 12% sodium dodecyl sulfate–polyacrylamide gels (SDS-PAGE), and its reactivity with anti-His-tag monoclonal antibody (Boster, Hubei, China) and mouse anti-*M. synoviae* polyclonal antibody were determined via western blotting. The concentration of rLP78 was determined using a BCA Protein Assay kit (Beyotime, China).

### Preparation of antisera against rLP78 in mice

Female BALB/c mice aged 8 weeks were subcutaneously injected with 30 μg of rLP78 emulsified with Montanide™ ISA 206 VG adjuvant (Seppic, Shanghai, China) at an equal ratio (w/w) at multiple points in the back. A booster dose was injected on days 14 and 28 after the first immunization. Blood samples were collected from the retro-orbital sinus after 2 weeks of the last booster immunization. A specific antibody against rLP78 in sera was evaluated via indirect ELISA (iELISA) and the ELISA titer was 1:100,000. The protocols were approved by the Ethics Committee of Animal Experimentation of Northwest A&F University (No. 220412).

### Conservation of LP78 in different *Mycoplasma synoviae* strains

The presence of the lp78 gene and its expression in other *M. synoviae* strains was investigated by PCR and western blotting. Briefly, *M. synoviae* strains W1, YL, HN, M6, and Q9 were harvested from broth cultures by centrifugation at 12,000 rpm and washed three times with PBS. The whole genome was extracted and applied to PCR amplification using lp78-1-F/ lp78-7-R primers. The PCR products were sequenced and then compared with *M. synoviae* strain MS-H.

For western blotting, the whole cell lysates were separated via SDS-PAGE (12% gels) and transferred to a PVDF membrane. The membrane was blocked with 10% skim milk in TBS (0.02 mol/L Tris, 0.15 mol/L NaCl, pH 7.4) containing 0.05% Tween-20 (TBST) for 2 h at room temperature. After washing with TBST, the membrane was incubated with mouse anti-rLP78 polyclonal antibody (1:500), followed by HRP-conjugated goat anti-mouse antibody (1:10,000) (Boster, Hubei, China). The signals were developed with an enhanced chemiluminescence (ECL) reagent (Millipore, Billerica, MA, United States).

### Subcellular localization of LP78 in *Mycoplasma synoviae*

To determine the distribution of LP78 in *M. synoviae*, a western blotting and an indirect immunofluorescence assay (IFA) were performed. For western blotting, the membrane and cytoplasmic proteins of the *M. synoviae* strain W1 were extracted using a protein extraction kit (Beyotime, China) according to the manufacturer’s instructions. The proteins were separated via SDS-PAGE (12% gels) and transferred to a PVDF membrane. The membrane was blocked with 10% skim milk in TBS (0.02 mol/L, pH 7.4) containing 0.05% Tween-20 (TBST) for 2 h at room temperature. The membrane was washed thrice with TBST and incubated with mouse anti-rLP78 polyclonal antibody (1:500) for 2 h at room temperature. Subsequently, it was washed thrice with TBST and incubated with HRP-conjugated goat anti-mouse antibody (1:10,000) (Boster, Hubei, China) at room temperature for 2 h. The protein band was visualized using an enhanced chemiluminescence (ECL) reagent (Millipore, Billerica, MA, United States).

For IFA, *M. synoviae* strain W1 was grown in modified Frey’s medium at 37°C with 5% CO_2_. At the log-phase, *M. synoviae* cells were harvested and washed three times with PBS. Afterwards, the cells were incubated with a 1:200 dilution of mouse anti-rLP78 polyclonal antibody or pre-immune serum overnight at 4°C. After washing, the cells were incubated with 1:500 dilution of FITC-labeled goat anti-mouse IgG (Boster, Hubei, China) for 1 h, and then visualized under a fluorescence microscope (Zeiss, Jena, Germany).

### Inhibition of *Mycoplasma synoviae* adherence to cells by mouse anti-rLP78 serum

For the adherence inhibition assay, *M. synoviae* W1 strain cells (1 × 10^7^ CCU/ml) were washed three times with PBS, and then incubated with mouse anti-rLP78 or nonimmune serum (1:20 dilution) at 37°C for 1 h. DF-1 cells were seeded into 24-well cell plates and grown to a confluent monolayer. After washing three times with PBS, DF-1 cells were blocked with 5% bovine serum protein (BSA) at 37°C for 30 min. Subsequently, *M. synoviae* cells pretreated with serum were added to the wells and incubated at 37°C for 1 h. The unbound *M. synoviae* cells were removed by washing three times with PBS. Then, the DF-1 cells were digested with 0.25% trypsin, followed by bacterial genome extraction using the TIANamp Bacteria DNA Kit (Tiangen, Beijing, China). The amounts of *M. synoviae* were determined by quantitative real-time PCR using primers targeting the 16S rRNA gene (217 bp) as described previously (Raviv and Kleven, 2009). The DNA copies were calculated according to the standard curve plotting the Ct values against 10-fold serial dilutions of the standard plasmid.

### Adhesion of rLP78 to host cells

An indirect immunofluorescence assay (IFA) was used to determine the adhesion of rLP78 to DF-1 cells. Briefly, DF-1 cells were cultured in a 96-well cell culture dish in DMEM medium for 24 h, and then fixed with 4% paraformaldehyde. After blocking with 5% BSA, the cells were incubated with 10 μg of rLP78 for 1 h. After washing to remove nonadherent proteins, the bound protein was stained with anti-His-tag monoclonal antibody (1:1,000 dilution) (Boster, Hubei, China) at 37°C for 1 h, followed by FITC-labeled goat anti-mouse IgG (1:500 dilution) (Boster, Hubei, China) for 1 h. Finally, the cell nuclei were stained with DAPI, and the immunofluorescence was detected using a fluorescence microscope (Zeiss, Jena, Germany). For the adherence inhibition assays, 100 μg/mL of rLP78 was incubated with mouse anti-rLP78 serum, or nonimmune serum (1:10 dilution) at 37°C for 1 h. Then, the mixtures were added to the fixed DF-1 cells as described above.

### Binding of rLP78 to cell membrane proteins

The ability of rLP78 to bind cell membrane proteins were determined by a microtiter plate adhesion assay (MPAA) and western blotting as previously described (Li et al., 2022; Peng, et al., 2023). For MPAA, membrane and cytoplasmic proteins were extracted from 2 × 10^7^ DF-1 cells using a commercial kit (Beyotime, China) according to the manufacturer’s instructions. Microtiter ELISA plate was coated with 5 μg/mL of cell membrane proteins, cytoplasmic proteins, or BSA diluted in sodium carbonate buffer (pH 9.6) and incubated overnight at 4°C. After blocking with 5% skim milk in PBST for 2 h at 37°C, different concentrations of rLP78 (ranging from 0.78 μg/mL to 100 μg/mL) were added and incubated for 2 h at 37°C. Unbound proteins were removed by washing with PBST, and adherence was evaluated by adding 100 μL of anti-His-tag monoclonal antibody (1:1,000 dilution) (Boster, Hubei, China) followed by 100 μL of HRP-conjugated goat anti-mouse IgG (1:10,000 dilution) (Boster, Hubei, China). Finally, the optical density (OD) values of the solutions were measured at 450 nm. For the adherence inhibition assay, 100 μg/mL of recombinant protein was mixed with mouse anti-rLP78 serum at various dilutions (ranging from 1:10 to 1:2,560) and incubated for 1 h at 37°C before being added to the wells.

For western blotting, the DF1 cell membrane and cytosolic proteins were separated by 12% SDS-PAGE and then transferred onto PVDF membranes. After blocking with 5% skim milk, the membrane was incubated with rLP78 (20 μg/mL) overnight at 4°C. After washing three times with TBST, the membrane was incubated with a 1:500 dilution of mouse anti-rLP78 polyclonal antibody at 37°C for 2 h. After washing three times with TBST, a 1:10,000 dilution of HRP-conjugated goat anti-mouse IgG (Boster, Hubei, China) was added and incubated at 37°C for 1 h.

### Binding activities of rLP78 to human fibronectin (hFn) and human plasminogen (hPlg)

The binding activities of rLP78 to hFn or hPlg were determined by ELISA and western blotting. For ELISA analysis, microtiter ELISA plates were coated with 100 μL of hFn or hPlg (Sigma-Aldrich, Burlington, MA, United States) at 5 μg/mL, and incubated overnight at 4°C. After blocking with 5% skim milk, 100 μL of rLP78 at different concentrations (ranging from 1.56 μg/mL to 100 μg/mL) was added and incubated for 2 h at 37°C. After being washed with PBST, the plates were treated with 100 μL of anti-His-tag monoclonal antibody (1:1,000 dilution) (Boster, Hubei, China), followed by 100 μL of HRP-conjugated goat anti-mouse IgG (1:10,000 dilution) (Boster, Hubei, China). Finally, the optical density (OD) values of the solutions were measured at 450 nm.

For western blotting, rLP78 was separated by 12% SDS-PAGE and transferred onto PVDF membranes. After blocking with 10% skim milk, the membrane was incubated with 5 μg/mL hFn or hPlg, followed by incubation with rabbit anti-fibronectin antibody or rabbit anti-plasminogen antibody (1:1,000 dilution) (Boster, Hubei, China) as the primary antibody, and HRP-conjugated goat anti-rabbit IgG (1:10,000 dilution) (Boster, Hubei, China) as the secondary antibody.

### Sensitivity, specify, and reproducibility of rLP78-based iELISA

For the development of rLP78-based iELISA, the optimal coating concentration of rLP78 and optimal dilution ratios of the tested sera were determined by a checkerboard titration method. The cut-off value of iELISA was determined by testing 83 serum samples collected from chickens that were free of *M. synoviae* infection. The S/P cut-off value was expressed as the mean S/P of 83 negative sera samples plus 3 standard deviations (SDs) to ensure 99% confidence for negative sera samples in this range.

To determine the sensitivity and specificity of rLP78-based iELISA, a total of 170 serum samples collected from chickens with known *M. synoviae* infection status were detected using both rLP78-based iELISA and a commercial ELISA kit (IDEXX, Westbrook, Maine, USA). The sensitivity and specificity of rLP78-based iELISA were calculated relative to those of the commercial ELISA kit. To determine the lowest detection limit, three serum samples (HI titer, 1:128) were serially diluted 2-fold from 1:100 to 1:51,200, and cross-reactivity of rLP78-based iELISA with positive sera against other avian pathogens, including *M. gallisepticum*, *Hpg*, NDV, AIV, IBV, and IBDV, was measured.

The reproducibility of rLP78-based iELISA was evaluated by testing 10 serum samples (five positive and five negative samples). Within-run precision was assessed in three plates in one run, and between-run precision was assessed in three runs. The mean OD, SD, and coefficient of variation (CV) were calculated for each test.

### Detection of antibody response against *Mycoplasma synoviae* in experimentally infected and immunized chickens

To investigate the antibody response against *M. synoviae* in chickens, 135 sequential chicken serum samples were detected using rLP78-based iELISA and a commercial ELISA kit (IDEXX, Westbrook, Maine, United States). Among the 135 serum samples, 90 samples were collected from 10 chickens infected with *M. synoviae* W1 (1 × 10^8^ CCU/mL) via tracheal routine, whereas 45 serum samples were collected from 5 chickens immunized with the commercial inactivated *M. synoviae* vaccine (YBF-MS1 strain) (Yebio Bioengineering, co., Ltd. of Qingdao, China) via muscular routine. The samples were collected at 0, 3, 7, 10, 14, 21, 28, 42, and 60 days after challenge or immunization.

### Statistical analysis

Statistical analyses were performed using IBM SPSS Statistics 20 (Armonk, NY, United States). Values given in the text are the mean ± SD from the experiment. Graphs were prepared in GraphPad Prism 8.0 (San Diego, CA, United States).

## Results

### Bioinformatic analysis

The full length of *lp78* gene in *M. synoviae* strain MS-H was 2,310 bp. The ORF was predicted to encode a 770-amino-acid protein with a molecular mass of 84 kDa containing six Trp residues (positions 53, 86, 264, 415, 610, 645), all encoded by TGA codons. The LP78 protein sequence was predicted to possess a signal peptide (cleavage site between amino acid position 24 and 25), but lack a classical transmembrane domain, and be an outer membrane protein. Comparison of the LP78 protein sequences in the databases found identities of 98.7% with strain 53 (AAZ43746.2), 97.4% with strain WVU1853 (AAZ43746.2), native strains SD2 (UZW64787.1), and HN01 (QGL45032.1), and 99.3% with native strains FJ-01 (QXV99750.1) and ZX313 (UZW64085.1). LP78 was predicted to have an antigenicity score of 0.643 (the threshold score for antigenicity is 0.4), and an adhesion score of 0.785 (the threshold score for adhesin is 0.51).

### Purification and identification of rLP78

**Figure 1:**
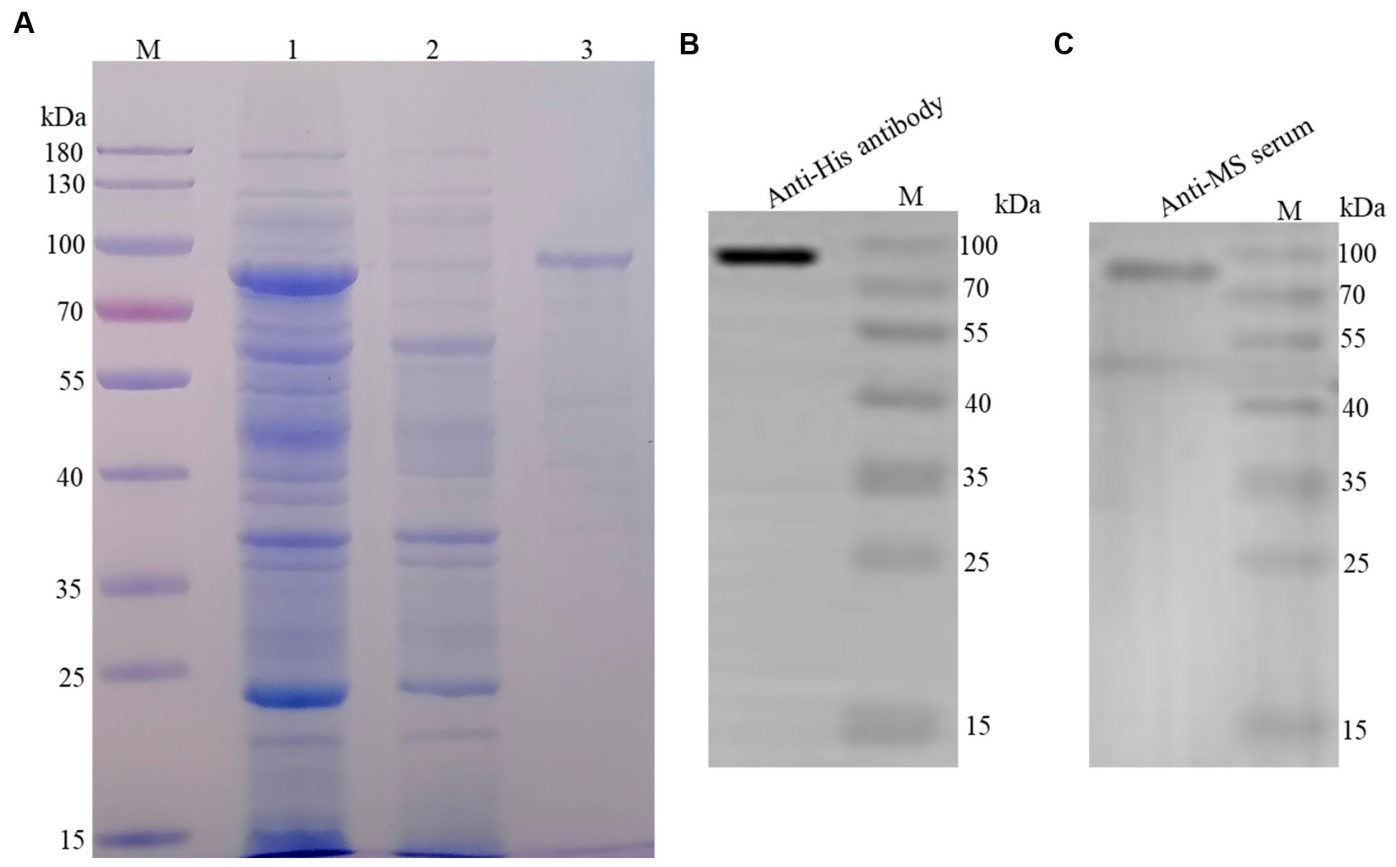
Purification and identification of rLP78. **(A)** SDS-PAGE analysis of the purification of rLP78 by Ni^+^ column. Lane M, protein molecular weight marker; Lane 1, the supernatant of the whole bacterial lysate; Lane 2, elution with 50 mM of imidazole; Lane 3, elution with 500 mM of imidazole. **(B)** Western blotting analysis of the purified rLP78 using anti-His-tag monoclonal antibody. **(C)** Western blotting analysis of the purified rLP78 using mouse anti-*M. synoviae* polyclonal antibodies.

The recombinant protein was purified using the Ni-NTA His-Bind^®^ Resin column and a band with a molecular weight of 80 kDa was observed in the elution (Figure 1A). In addition, the recombinant protein was quantified via western blotting using an anti-His-tag monoclonal antibody (Figure 1B) and mouse anti-*M. synoviae* sera (Figure 1C). The concentration of the recombinant protein was estimated to be 1.5 mg/mL.

### Conservation of LP78 in different *Mycoplasma synoviae* field strains

**Figure 2:**
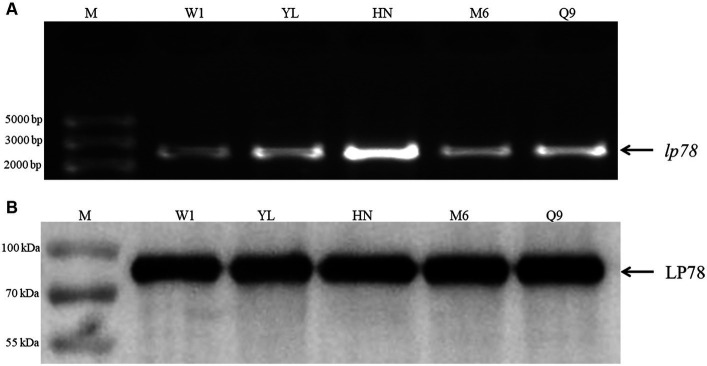
Identification of LP78 in different *M. synoviae* strains. **(A)** Amplification of lp78 gene in different *M. synoviae* strains. Genomic DNA was extracted from *M. synoviae* cultures and used as templates. **(B)** Immunoblot analysis of the expression of LP78 in different *M. synoviae* strains. Whole cell protein from different *M. synoviae* strains cells were separated by 12% SDS-PAGE and transferred to a PVDF membrane. The membrane was incubated with mouse anti-rLP78 polyclonal antibody, followed by HRP-conjugated goat anti-mouse antibody.

The conservation of LP78 was identified by PCR and western blotting. As shown in Figure 2A, a fragment of 2,235 bp was detected from the five *M. synoviae* field strains by PCR. After sequencing, a high identity (98.7–97.4%) was found between the five *M. synoviae* strains and strain MS-H. In addition, an 80-kDa protein band was specifically recognized by mouse anti-rLP78 polyclonal antibodies in the five strains (Figure 2B). The bands were in accordance with the theoretical molecular mass of rLP78, which indicated the LP78 was expressed by all strains examined.

### Subcellular localization of LP78 in *Mycoplasma synoviae*

**Figure 3:**
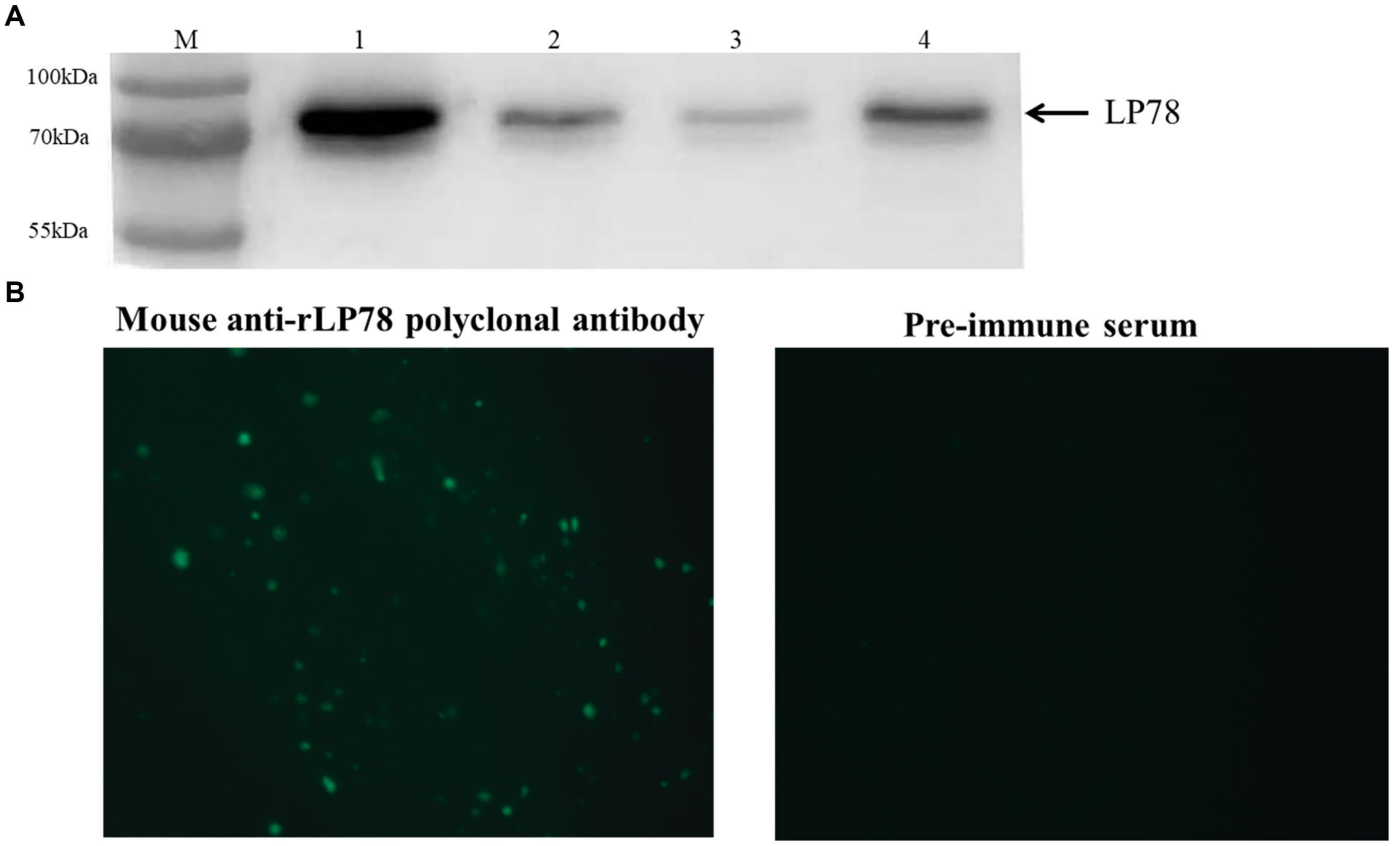
Subcellular localization of LP78 in *M. synoviae* by western blotting and IFA using mouse anti-rLP78 serum. **(A)** Western blotting analysis. Lanes 1: rLP78; Lane 2: Cytosolic proteins; Lane 3: Membrane proteins; Lane 4: Total cellular proteins. **(B)** IFA of *M. synoviae* cells incubated with mouse anti-rLP78 polyclonal antibody and pre-immune serum.

Western blotting and IFA was performed to determine the distribution of LP78 in *M. synoviae* using mouse anti-rLP78 polyclonal antibody. Western blotting showed that LP78 was found to be located both on the membrane and in the cytoplasm of *M. synoviae* (Figure 3A). In particular, a stronger band was observed for cytoplasmic proteins than for membrane proteins, indicating that LP78 was primarily located in the cytoplasm of *M. synoviae*. IFA demonstrated that *M. synoviae* cells stained with fluorescein isothiocyanate when incubated with mouse anti-rLP78 polyclonal antibody, but no when incubated with pre-immune serum (Figure 3B).

### Adherence inhibition of *Mycoplasma synoviae* to DF-1 cells using antibodies against rLP78

**Figure 4:**
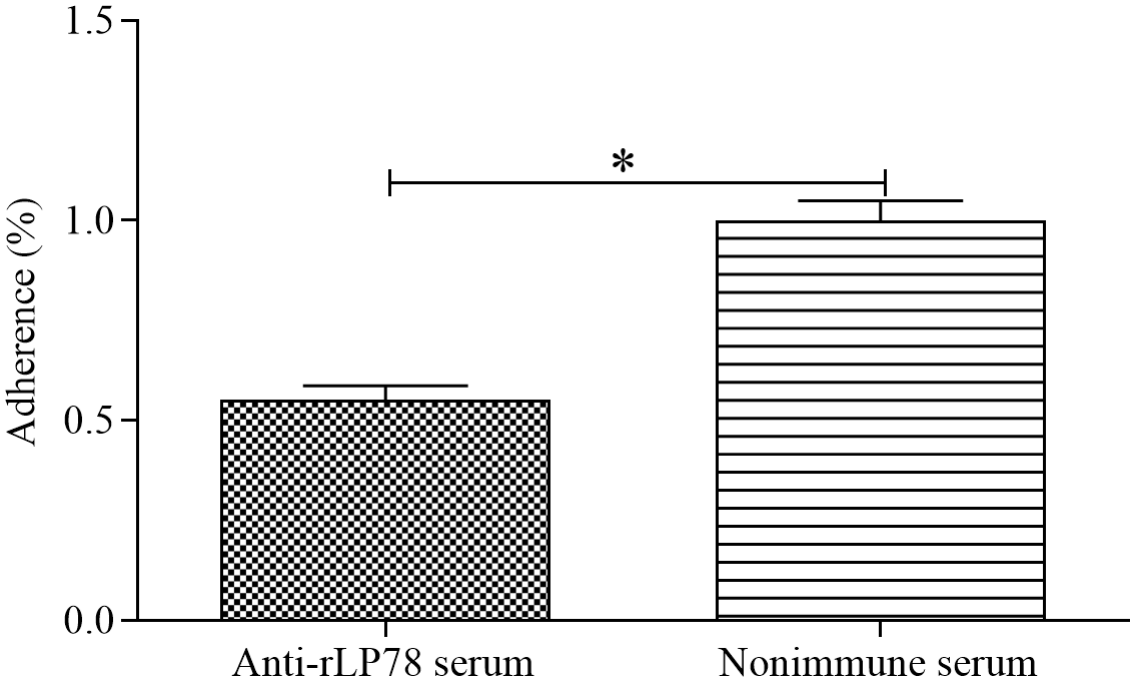
Adherence inhibition of *M. synoviae* to DF-1 cells by anti-rLP78 serum. *M. synoviae* cells were pre-incubated with anti-rLP78 serum, and then the mixtures were added to DF-1 cells. The amounts of bound *M. synoviae* were determined by qPCR. Adhesion rate: number of *M. synoviae* in the cells incubated with anti-rLP78 serum/number of *M. synoviae* in the cells incubated with nonimmune serum. Data are expressed as the mean ± SD of samples in triplicate. *p < 0.05.

The antibody inhibition assay was carried out to assess the contribution of LP78 in *M. synoviae* cytoadhesion. Compared with the nonimmune serum group, the number of *M. synoviae* attached to DF-1 cells was greatly reduced after treatment with anti-rLP78 serum (*p* < 0.05) (Figure 4). The result implied that LP78 plays an important part in *M. synoviae* adherence to DF-1 cells.

### Adherence of rLP78 to DF-1 cells

**Figure 5:**
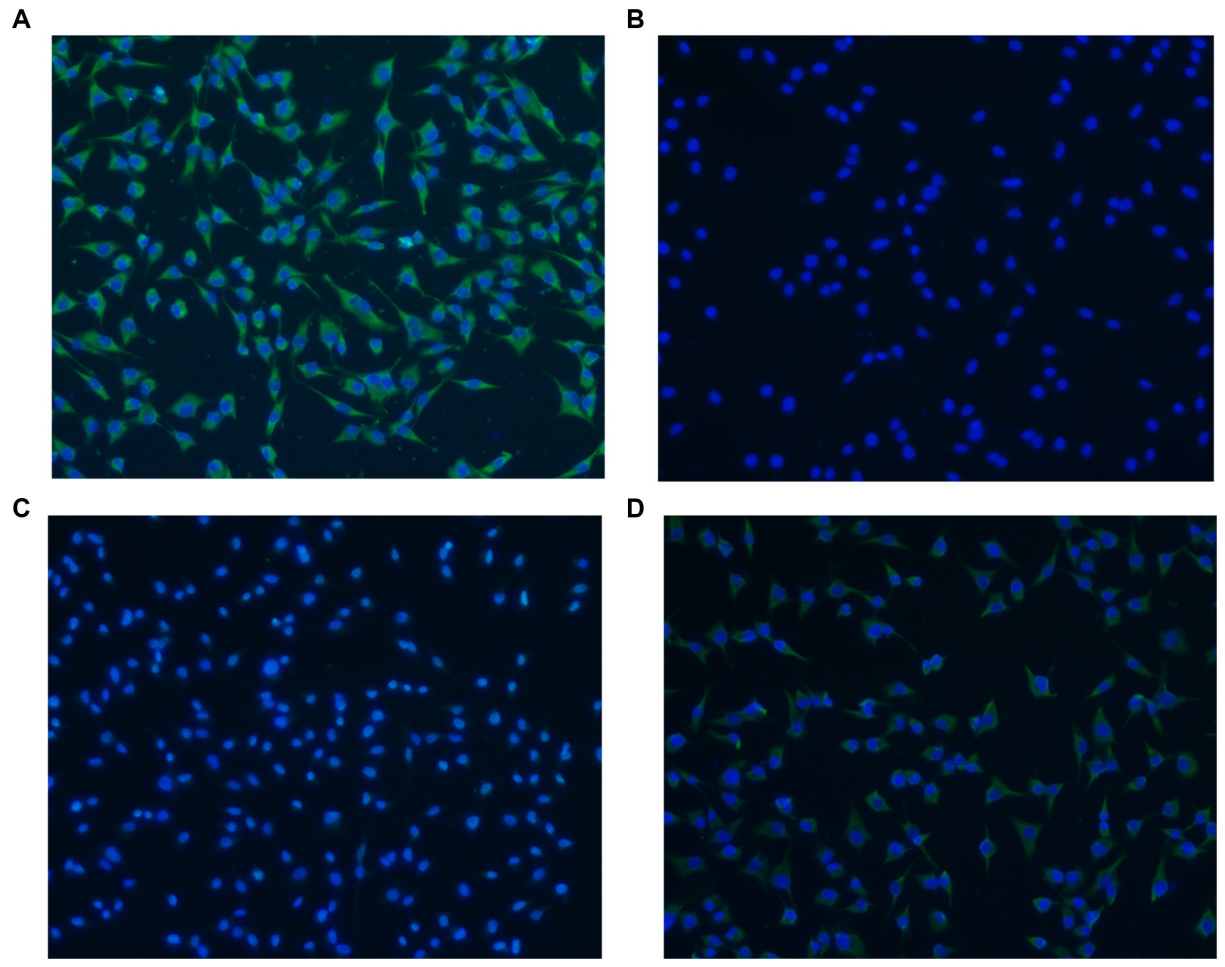
Adherence of rLP78 to DF-1 cells detected by IFA. DF-1 cells were incubated with rLP78 **(A)** or pET-28a control protein **(B)**. Bound proteins were detected by an anti-His-tagged monoclonal antibody and immunostained with FITC-conjugated goat anti-mouse IgG. **(C)** Mouse anti-rLP78 serum inhibited rLP78 adherence. **(D)** Nonimmunized mouse serum showed no inhibition of rLP78 adherence to DF-1 cells. Cell nuclei were labeled with 4,6-diamidino-2-phenylindole (DAPI).

The adherence of rLP78 to DF-1 cells was visualized by IFA. As shown in Figure 5A, rLP78 was able to adhere to DF-1 cells. No adherence was observed for the control (Figure 5B). Additionally, rLP78 adhered to DF-1 cells was effectively inhibited by mouse anti-rLP78 serum (Figure 5C), whereas nonimmunized mouse serum did not inhibit adherence to DF-1 cells (Figure 5D).

### Binding of rLP78 to cell membrane proteins

**Figure 6:**
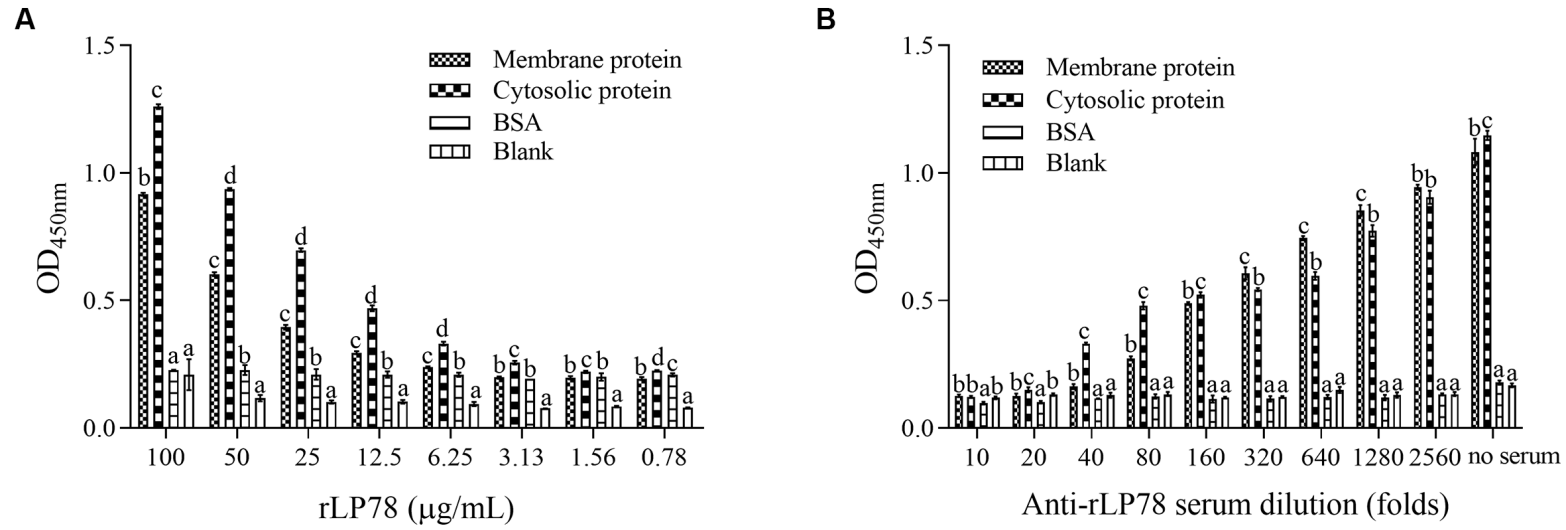
Activity of rLP78 to adhere to membrane proteins of DF-1 cells. Microtiter ELISA plates were coated with the extracted membrane protein, cytosolic protein or BSA. **(A)** Different concentrations of rLP78 were added to individual wells. Bound proteins were detected using a mouse anti-His-tag monoclonal antibody. **(B)** Adherence of rLP78 (100 μg/mL) to membrane proteins of DF-1 cells was inhibited by mouse anti-LP78 serum and further detected using goat anti-mouse IgG. Bars represent the mean ± standard deviation of the OD_450nm_ values of samples in triplicate. The differences were compared between each group. The same letter indicates no obvious difference (*p* > 0.05); different letters indicate a significant difference (*p* < 0.05).

**Figure 7:**
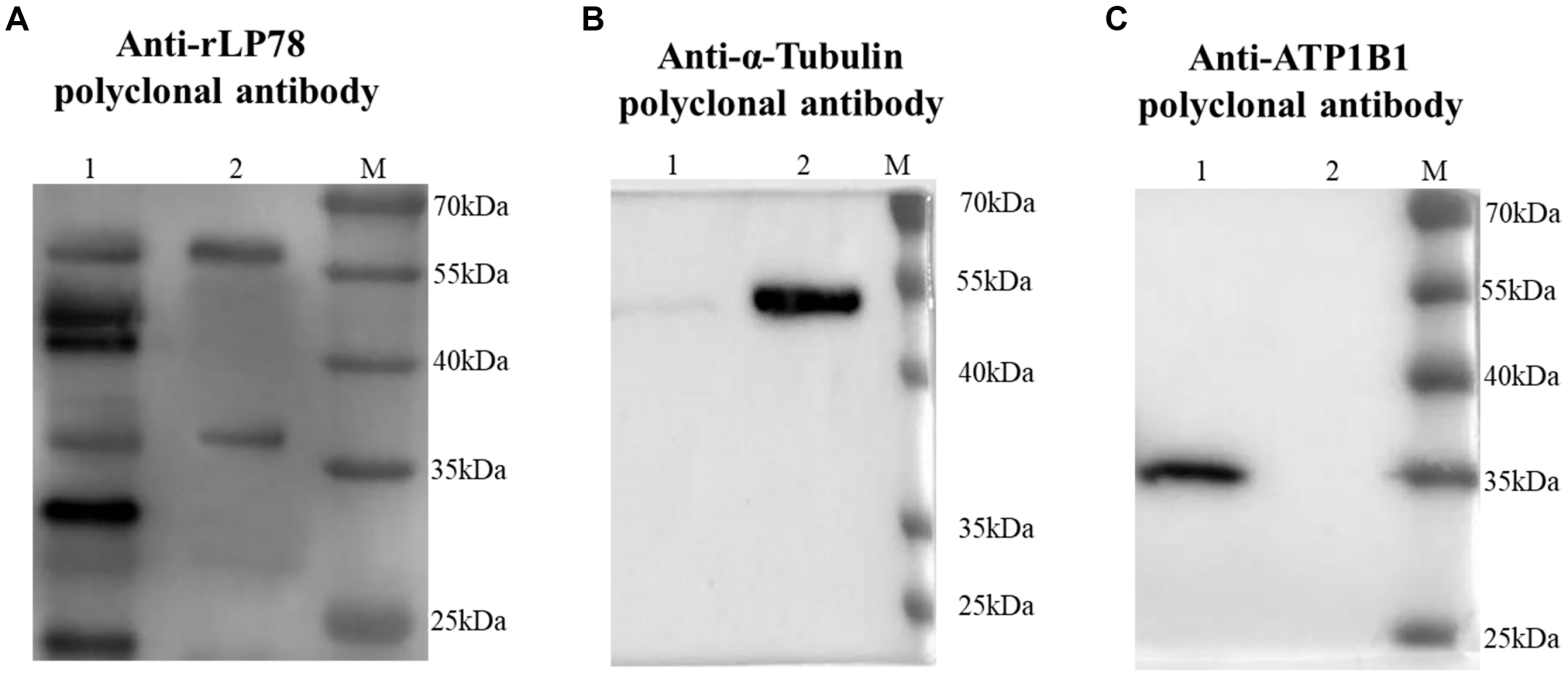
Screening of DF1 cells membrane proteins that bound to rLP78 by western blotting. DF1 cells cytosolic protein (lane 1) and cells membrane protein (lane 2) were transferred to PVDF membranes and incubated with rLP78 (20 μg/mL). Subsequently, the membranes were incubated with mouse anti-rLP78 polyclonal antibody **(A)**, rabbit anti-α-Tubulin polyclonal antibody **(B)**, and rabbit anti-ATP1B1 polyclonal antibody **(C)**.

The binding activity of rLP78 to DF-1 cells was quantitatively determined by MPAA and western blotting. As shown in Figure 6A, rLP78 bound to the membrane and cytosolic proteins of DF-1 cells in a dose-dependent manner from 6.25 μg/mL to 100 μg/mL (*p* < 0.05). The binding was significantly inhibited by anti-rLP78 serum in a dose-dependent manner (Figure 6B) (*p* < 0.05). When the antibody levels decreased, the OD_450nm_ values gradually increased. In the membrane protein incubated with mouse anti-rLP78 polyclonal antibody, two bands were observed: one between 35 kDa and 40 kDa and the other between 55 kDa and 70 kDa (Figure 7A). The two bands were also found in the cytosolic protein (Figure 7A). The DF1 cell membrane and cytosolic proteins were successfully extracted, with no contamination from other fractions (Figures 7B,C).

### Binding ability of rLP78 to hFn and hPlg

**Figure 8:**
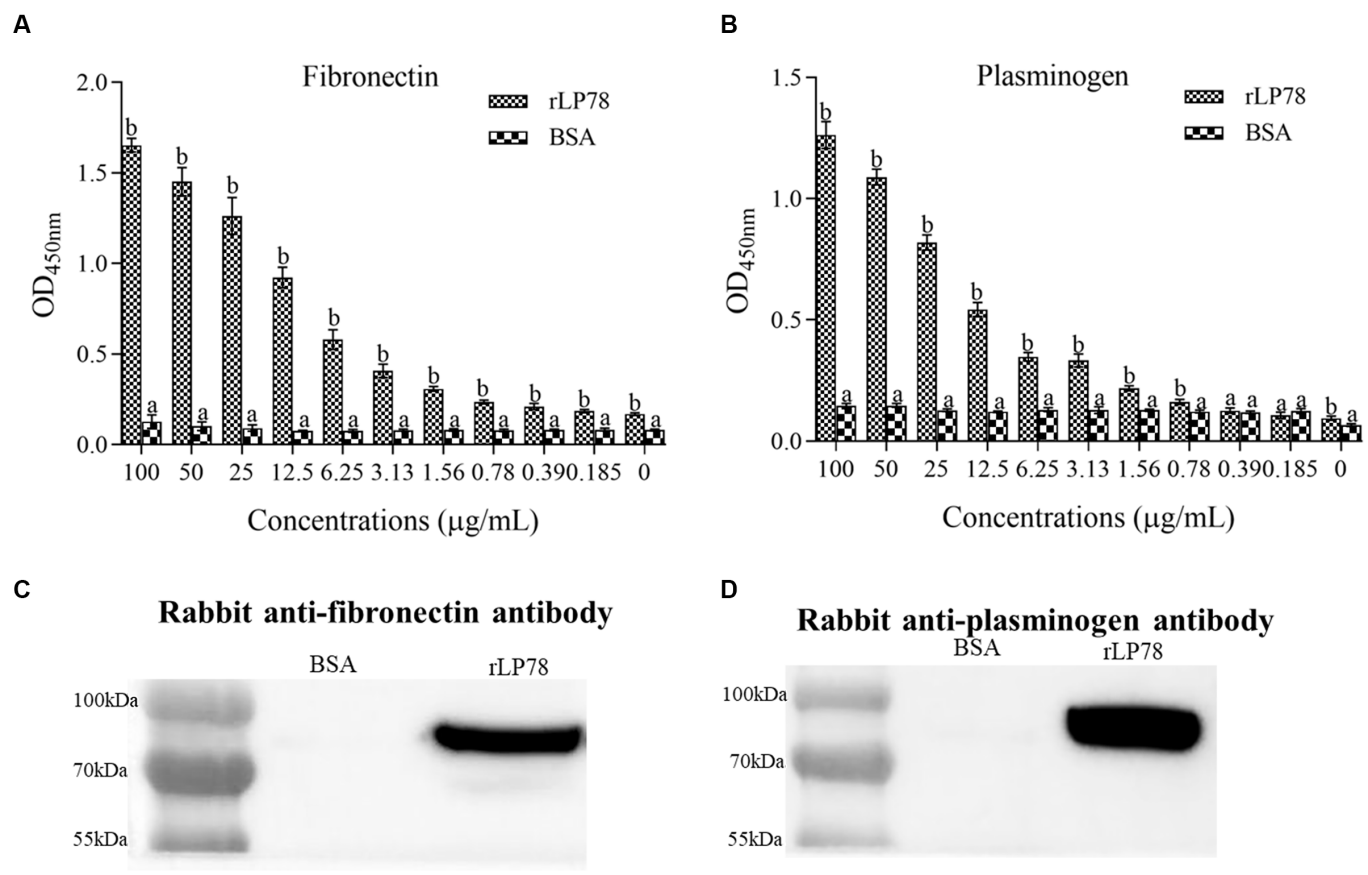
Binding ability of rLP78 to human fibronectin (hFn) and human plasminogen (hPlg). The binding ability of rLP78 to hFn **(A)** or hPlg **(B)** was identified by ELISA. The plates were coated with 5 μg/mL of hFn or hPlg. Different concentrations of rLP78 or BSA were added to individual wells. Bound proteins were detected using a mouse anti-His-tag monoclonal antibody. Bars represent the mean ± standard deviation of the OD values of samples in triplicate. The differences were compared between each group. The same letter indicates no obvious difference (*p* > 0.05); different letters indicate a significant difference (*p <* 0.01). Binding ability of rLP78 to hFn **(C)** or hPlg **(D)** was confirmed by western blotting. Bound hFn was determined by rabbit anti-hFn antibody or rabbit anti-plasminogen antibody. BSA was chosen as negative control.

The binding ability of rLP78 to hFn and hPlg was determined by ELISA and western blotting. ELISA results showed that rLP78 bound to immobilized hFn (Figure 8A) or hPlg (Figure 8B) in a dose-dependent manner and the OD_450nm_ values of rLP78 were significantly higher than BSA (*p* < 0.05). To further confirm the specific interaction between rLP78 and hFn or hPlg, western blotting was performed. After rLP78 was transferred to PVDF membranes, hFn or hPlg was added. The bound hFn or hPlg was detected with rabbit anti-fibronectin antibody (Figure 8C) or rabbit anti-plasminogen antibody (Figure 8D) at the site corresponding to the band of purified rLP78. No obvious interactions between hFn or hPlg and BSA were observed.

### Sensitivity, specificity, and reproducibility of rLP78-based iELISA

The checkerboard titration results showed that the optimal coating concentration of rLP78 was 1 μg/mL and the optimal dilution ratios of the tested sera were 1:600 for rLP78-based iELISA (Supplementary Figure S3). The cut-off value of iELISA was calculated to be 0.341. Serum samples from infected or immunized chickens were considered positive for *M. synoviae* infection when the S/*p* value was ≥0.341; otherwise, they were considered negative.

**Figure 9:**
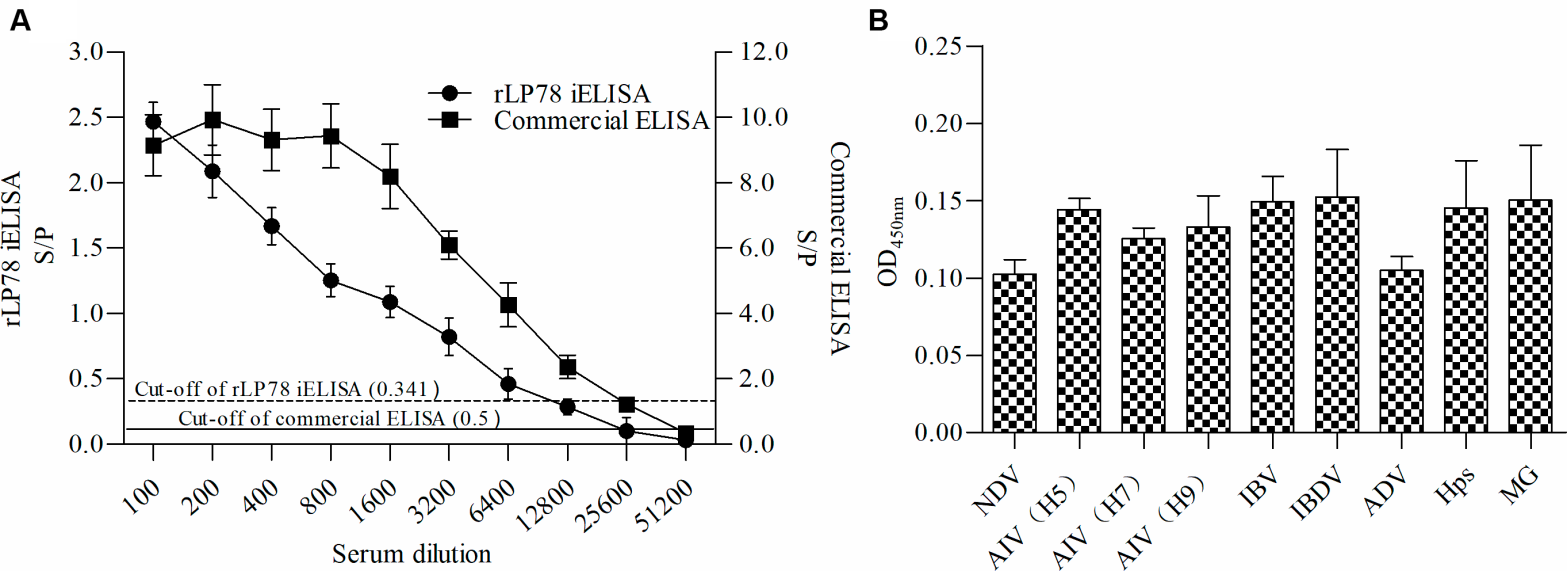
Sensitivity and specificity of rLP78 iELISA. **(A)** Determination of the largest dilution of positive chicken serum for anti-*M. synoviae* antibodies. The HI titer of chicken anti-*M. synoviae* serum was 1:128. **(B)** The cross-reactivity of rLP78 iELISA with antibodies against other avian pathogens, including NDV, AIV (H5, H7, H9), IBV, IBDV, ADV, *Hps*, and MG. Bars represent the mean ± standard deviations of the S/*p* values or OD_450nm_ values of samples (*n* = 3). The full line and dashed line represent the cut-off of rLP78-based iELISA (S/*p* ≥ 0.341 is positive, otherwise, is negative) and commercial ELISA (S/*p* > 0.5 is positive, otherwise, is negative), respectively.

The sensitivity and specificity of rLP78-based iELISA and a commercial ELISA kit were compared in 170 clinical serum samples. Of the 119 positive serum samples identified using the commercial kit, 102 samples were classified as positive using rLP78-based iELISA (Table 1). Of the 51 negative serum samples identified using the commercial kit, 48 samples were classified as negative using rLP78-based iELISA (Table 1). The results of rLP78-based iELISA and the commercial ELISA kit were inconsistent in 20 samples. Therefore, the total agreement between rLP78-based iELISA and the commercial ELISA kit was 88.2%, with the sensitivity and specificity of rLP78-based iELISA being 85.7 and 94.1%, respectively. For different dilutions of positive sera (HI titer, 1:128), positive reactions were observed at the dilution of 1:6,400 in rLP78-based iELISA and at the dilution of 1:12,800 or 1:25,600 in the commercial kit (Figure 9A).

**Table 1:** Determination of sensitivity and specificity of rLP78 iELISA.

		IDEXX ELISA		Sensitivity(%)	Specificity (%)	Accuracy(%)
		Positive	Negative			
rLP78 ELISA	Positive	102	3	85.7% (102/119)		
	Negative	17	48		94.1% (48/51)	
	Total	119	51			88.2% (150/170)

To verify the cross-reactivity of rLP78-based iELISA, it was used to detect sera against *M. gallisepticum*, *Hpg*, AIV, NDV, IBV, and IBDV. All samples yielded a negative result, indicating that rLP78-based iELISA had no cross-reactivity with the tested sera (Figure 9B). The reproducibility of rLP78-based iELISA was determined by calculating the CV of the OD values of 10 serum samples. The inter-assay CV of the 10 samples ranged from 4.2 to 6.8%, whereas the intra-assay CV ranged from 3.1 to 8.9%.

### Antibody response against *Mycoplasma synoviae* in experimentally infected and immunized chickens

**Figure 10:**
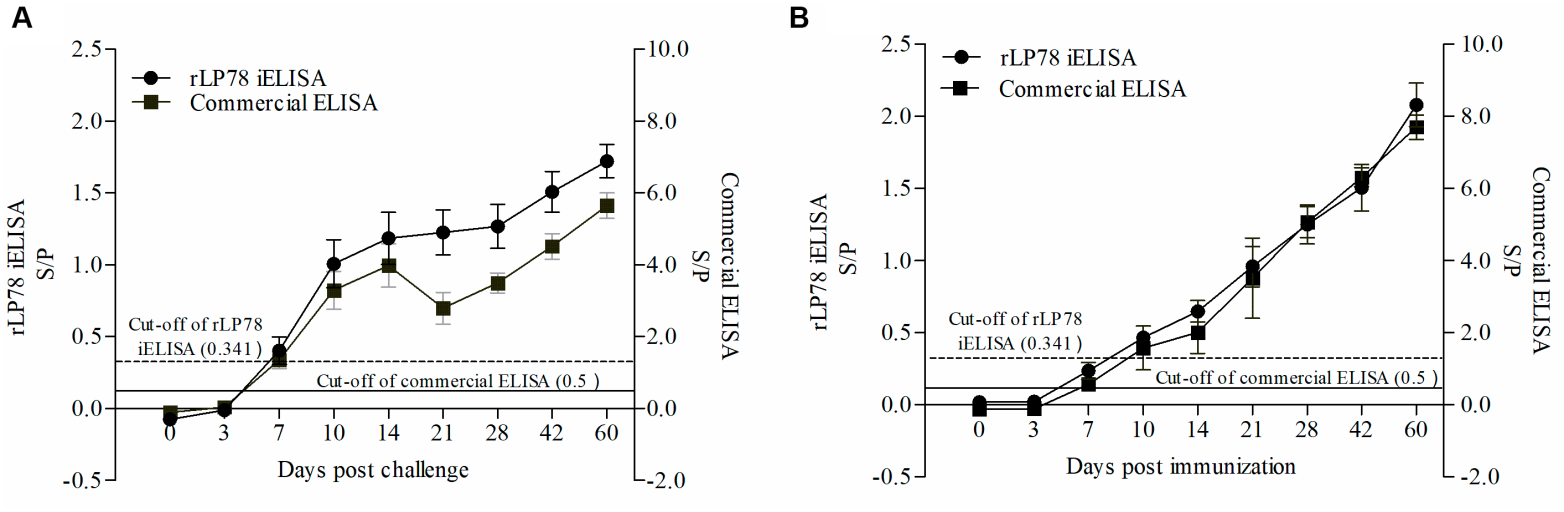
Detection of antibody response against *M. synoviae*. **(A)** Serum samples were collected from 10 chickens infected by *M. synoviae* at 0–60 days after inoculation. **(B)** Serum samples were collected from 5 chickens immunized with inactivated *M. synoviae* vaccine at 0–60 days after inoculation. The full line and dashed line represent the cut-off of rLP78-based iELISA (S/p ≥ 0.341 is positive, otherwise, is negative) and commercial kit (S/p > 0.5 is positive, otherwise, is negative), respectively. Bars represent the mean ± standard deviations of the S/p values of samples in each group.

To test the antibody response of iELISA, 90 infected serum samples and 45 immunized serum samples collected from chickens between 0 and 60 days were tested. The results of both rLP78-based iELISA and the commercial ELISA kit showed that the chickens seroconverted on day 7 after experimental infection, with a positive rate of 4/10 and 9/10, respectively (Figure 10A). All chickens seroconverted on day 10 after experimental infection and remained positive until day 60 after infection. In chickens immunized with the *M. synoviae* bacterin, the two ELISAs detected seroconversion on day 7 after immunization, with a positive rate of 1/5 for iELISA and 2/5 for the commercial kit (Figure 10B). Both ELISAs detected seroconversion at a positive rate of 5/5 on day 14 after immunization and remained positive until day 60 after immunization.

## Discussion

In recent years, the incidence of *M. synoviae* infection has increased and led to serious economic losses in the poultry industry worldwide. Attachment of mycoplasmas to host cells is a crucial step in colonization and subsequent infection, which is predominantly mediated by membrane proteins and lipoproteins (Lockaby et al., 1999; Browning et al., 2011). Various membrane-associated proteins, incuding vlhA, enolase, PdhA and PdhB subunits, PdhD and NADH oxidase, are involved in *M. synoviae* cytadhesion (Noormohammadi et al., 1997; Bao et al., 2014, 2021; Hu et al., 2022; Qi et al., 2022). *Mycoplasma* lipoproteins have been demonstrated to exert a variety of effects during infection and interaction with the hosts, including immunomodulatory, antigenic variation, transporter operation, and cytoadhesion (Athamna et al., 1997; Browning et al., 2011; Christodoulides et al., 2018). LP78 belongs to P80 family lipoprotein with undefined functions.

Comparison of the LP78 protein sequences in the databases revealed a high level of identity (97.4 to 99.3%). The conservation was confirmed by detecting LP78 among different native field strains. In addition, high levels of antibodies against LP78 were found in the *M. synoviae*-infected chicken serum and field serum through LP78-based iELISA. The findings imply LP78 plays an important role during infection. Bioinformatics analysis demonstrated that LP78 is located on the cell membrane surface of *M. synoviae* with a signal peptide but lack membrane-spanning domains. Western blotting and IFA results in the current investigation revealed that LP78 is exposed to the surface. Other mycoplasmas surface-exposed proteins were also shown to be lack of classical membrane-spanning domains (May et al., 2006; Guo et al., 2017; Chen et al., 2018). In addition, LP78 was also found in cytoplasmic fractions, it will be meaningful to investigate its functions in the cytoplasm. A signal peptide was found in LP78 by bioinformatics analysis, which suggests LP78 has secretory proteins characteristics. However, it has not been verified that LP78 is a secreted protein in a previous study (Rebollo Couto et al., 2012).

Although *M. synoviae* is mostly considered to be an extracellular pathogen, it can adhere to and subsequently invade erythrocytes, synovial sheath cells, and chicken embryonic fibroblast cells (Dusanic et al., 2009; Xu et al., 2020). Adherence of rLP78 to DF-1 cells was visualized by IFA, and the adherence was effectively inhibited by anti-rLP78 serum. The MPAA results showed that rLP78 bound to the membrane proteins of DF-1 cells in a dose-dependent manner and that the binding was inhibited by anti-rLP78 serum, further supporting the adherence. Furthermore, the adherence of *M. synoviae* to DF-1 cells was significantly reduced following pretreatment with anti-rLP78 serum. These provide additional evidences that LP78 contributes to the adhesion of *M. synoviae* to cells. However, anti-rLP78 serum only partially prevented adhesion to DF-1 cells. The fact that mycoplasmas cytadherence is a complex, multifactorial process involving numerous membrane proteins and cytoskeletal elements (Razin and Jacobs, 1992). In addition to LP78, other proteins have also been proven to contribute to the adhesion process of *M. synoviae*, such as vlhA, enolase, PdhA, PdhB, PdhD and NADH oxidase (Noormohammadi et al., 1997; Bao et al., 2014, 2021; Hu et al., 2022; Qi et al., 2022). It has been established that vlhA facilitates *M. synoviae* attachment to sialylated receptors on host cells (May et al., 2014). Further research should be carried out on the receptors that are mediated by LP78.

Although *M. synoviae* has been shown to invade host cells, its mechanism remains unclear. In previous studies, enolase, PdhA, PdhB, PdhD and NADH oxidase from *M. synoviae* have been identified as FnBPs and PlgBPs (Bao et al., 2014, 2021; Hu et al., 2022; Qi et al., 2022). The binding of FnBPs to Fn mediates not only the adherence of microorganism to extracellular matrices but also to the surface of host cells. It is now established that Fn can act as a molecular bridge between the FnBPs on the surface of microorganisms and the integrin on host cells (Henderson et al., 2011). This complex induces integrin clustering and triggers the actin cytoskeleton to be reorganized, which will assist in the internalization of microorganisms and facilitate cell invasion (Henderson et al., 2011). Additionally, the blocking of Fn significantly decreases the adherence of microorganisms to host cells (Yu et al., 2018). Apart from degradation of fibrin clot in fibrinolysis, plasmin degrades various ECMs and connective tissue components, including fibronectin and laminin (Bhattacharya et al., 2012). Traditionally, the activation pathway of Plg depends on tissue plasminogen activator (tPA) and urokinase (uPA). A few bacterial species, such as *streptococci*, can produce activator, which may be employed to activate the host Plg (Verhamme et al., 2015). In most bacterial species, Plg can be recruited from the surrounding environment to the bacterial surface and then promoted to be converted into plasmin (Raymond et al., 2015). In the present study, LP78 was identified to be PlgBPs. Whether the binding of LP78 would increase the susceptibility of Plg to be activated by tPA should be investigated further. The finding will contribute to understanding the mechanism of *M. synoviae* cell invasion.

Since LP78 is an immunodominant antigen found on the surface of *M. synoviae*, it may be used as a diagnostic antigen. Owing to its high sensitivity and ease of use, ELISA is an efficient alternative to SPA and HI tests for screening large numbers of serum samples. In a study, an ELISA was developed based on a protein fraction (p46–52) that was extracted from the Triton X-114 detergent phase and further purified via ion exchange chromatography (Gurevich et al., 1995). The results of this ELISA were highly consistent with those of the SPA test (Gurevich et al., 1995). A Dot-ELISA was developed based on the p41 antigen purified via SDS-PAGE, and was found to be more sensitive than HI (Avakian and Kleven, 1990). Although ELISA based on purified native antigens are sensitive, the production of sufficient and consistent antigens is expensive. Alternatively, an iELISA developed using recombinant MSPB protein expressed in *E. coli* showed good performance in the detection of anti-*M. synoviae* antibodies (Noormohammadi et al., 1999). In this study, we established and evaluated rLP78-based iELISA. Compared with a commercial ELISA kit, rLP78-based iELISA demonstrated less sensitivity in the detection of *M. synoviae*-positive serum samples. This result was consistent with that of the detection of different dilutions of *M. synoviae*-positive sera. Positive reactions were observed at the maximum dilution of 1:6,400 in rLP78-based iELISA and at the maximum dilution of 1:12,800 and 1:25,600 in the commercial ELISA kit. However, rLP78-based iELISA could detect seroconversion in 4/10 chickens on day 7 after experimental infection, with all chickens showing positive reactions between 10 and 60 days. These findings indicate that rLP78-based iELISA can be used in the diagnosis of early-stage *M. synoviae* infection. No cross-reactivity of rLP78-based iELISA was observed with other poultry pathogens, especially *M. gallisepticum* -positive serum samples. Therefore, LP78 is a good candidate for the development of diagnostic tests for *M. synoviae* infection. The prevalence of *M. synoviae* infection in Chinese commercial poultry farms was investigated using rLP78-based iELISA. The total seropositive rate of the tested samples was 58.77%, with the highest rate (74.91%) (Supplementary Table S2). A serological survey of *M. synoviae* infection was conducted in China during 2010–2015, and the seropositive rates in different provinces ranged from 24.70 to 57.20% (Xue et al., 2017). The results suggest that *M. synoviae* infection is a serious concern in China at present. In addition to routine serological monitoring, biosecurity, vaccination, and antibiotic administration are important measures for the control of *M. synoviae* infection (Kleven, 2008).

## Conclusion

In summary, the present study demonstrated that LP78 is a conservative, surface-exposed *M. synoviae* putative adhesion with Fn/Plg-binding activity, making it a good candidate for the development of diagnostic tests for *M. synoviae* infection.

## Data availability statement

Publicly available datasets were analyzed in this study. This data can be found at: https://www.ncbi.nlm.nih.gov/nuccore/; CP021129.

## Ethics statement

The animal study was approved by the Ethics Committee of Animal Experimentation of Northwest A&F University. The study was conducted in accordance with the local legislation and institutional requirements.

## Author contributions

SH: Data curation, Formal analysis, Investigation, Writing – original draft. YW: Data curation, Formal analysis, Software, Writing – review & editing. LW: Supervision, Writing – review & editing. WC: Data curation, Formal analysis, Investigation, Writing – review & editing. BW: Supervision, Writing – review & editing. JF: Data curation, Formal analysis, Investigation, Writing – review & editing. XH: Data curation, Writing – review & editing. XQ: Supervision, Writing – review & editing. JW: Conceptualization, Funding acquisition, Project administration, Writing – review & editing.

## Footnotes

^1^https://web.expasy.org/protscale/

^2^https://blast.ncbi.nlm.nih.gov/

^3^https://services.healthtech.dtu.dk/services/SignalP-5.0/

^4^https://services.healthtech.dtu.dk/services/TMHMM-2.0/

^5^http://cello.life.nctu.edu.tw/

^6^http://www.ddg-pharmfac.net/vaxijen/VaxiJen/VaxiJen.html

^7^https://violinet.org/vaxign/
