# Supplementary material for: Mycoplasma synoviae LP78 is a fibronectin/plasminogen binding protein, putative adhesion, and potential diagnostic antigen
Source: Front Microbiol. 2024 Jan 9;14:1335658. doi: 10.3389/fmicb.2023.1335658 (PMC10803467; doi:10.3389/fmicb.2023.1335658)
Supplement: Supplementary file 1 [file Data_Sheet_1.docx]

**Supplementary figures**


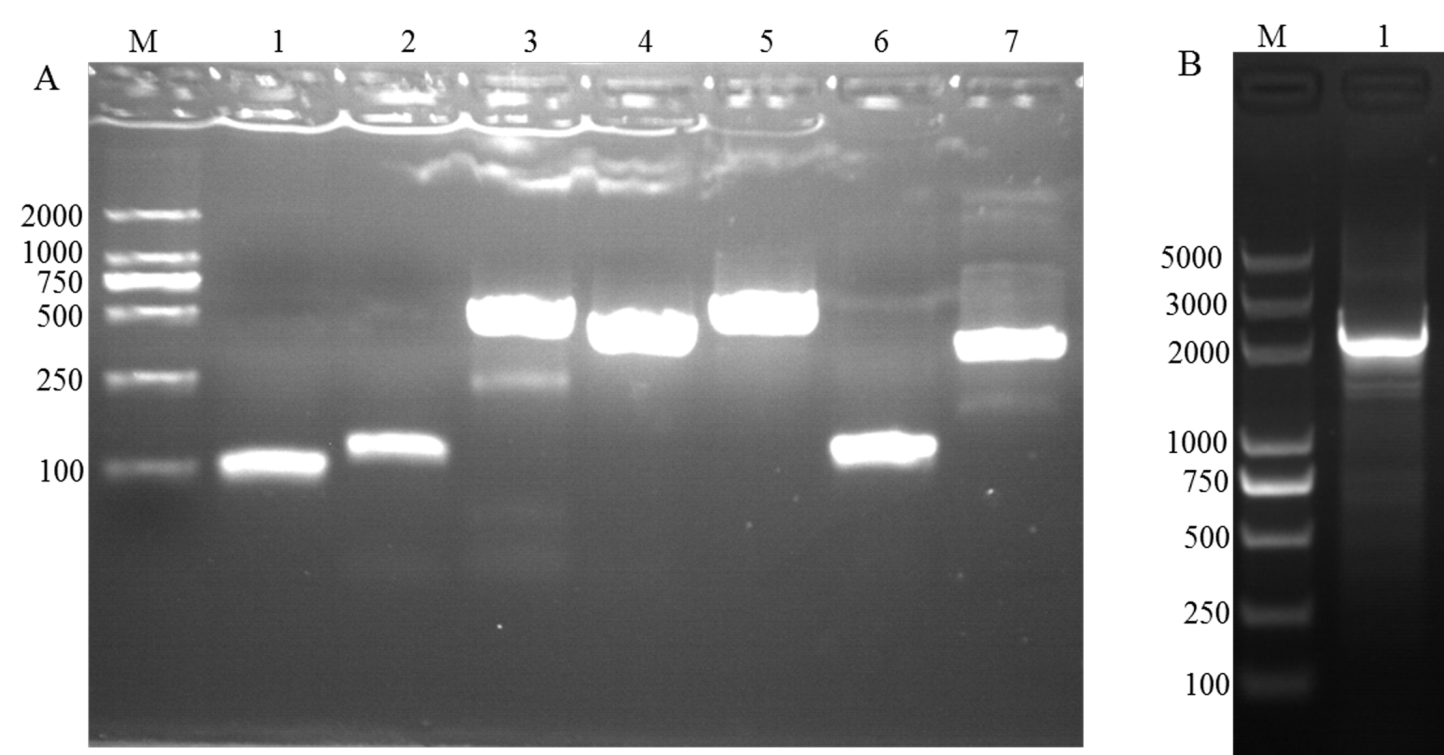


**Supplementary FIGURE S1** **Electrophoresis gel demonstrating PCR products of mutations and ligation of the *lp78* gene**. (**A**) PCR products of mutations: Lane 1 to Lane 7 were the PCR products of mutation, as expected, the sizes were 99 bp, 126 bp, 552 bp, 482 bp, 611 bp, 129 bp and 398 bp; Lane M: Nucleotide marker. (**B**) Full length of *lp78* gene: Lane M: Nucleotide marker; Lane 1 was the PCR product obtained using the seven purified fragments as template.


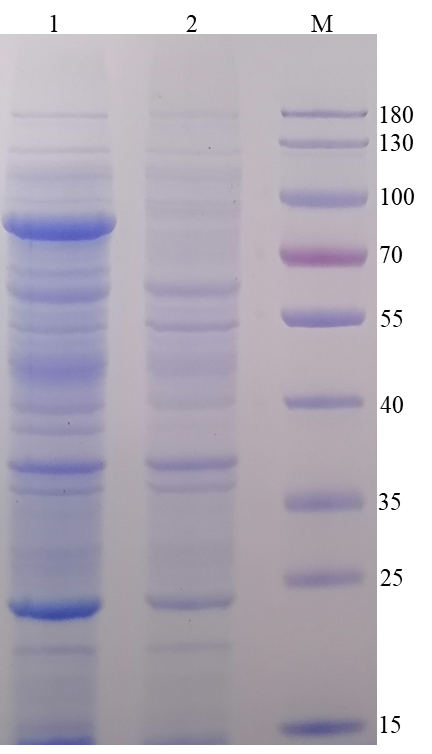


**Supplementary FIGURE S2. SDS-PAGE analysis of the soluble of rLP78 expressed in BL21 (DE3)**. Lane M, protein molecular weight marker; Lane 1, the supernatant of the whole bacterial lysate; Lane 2, the pellet of the whole bacterial lysate.


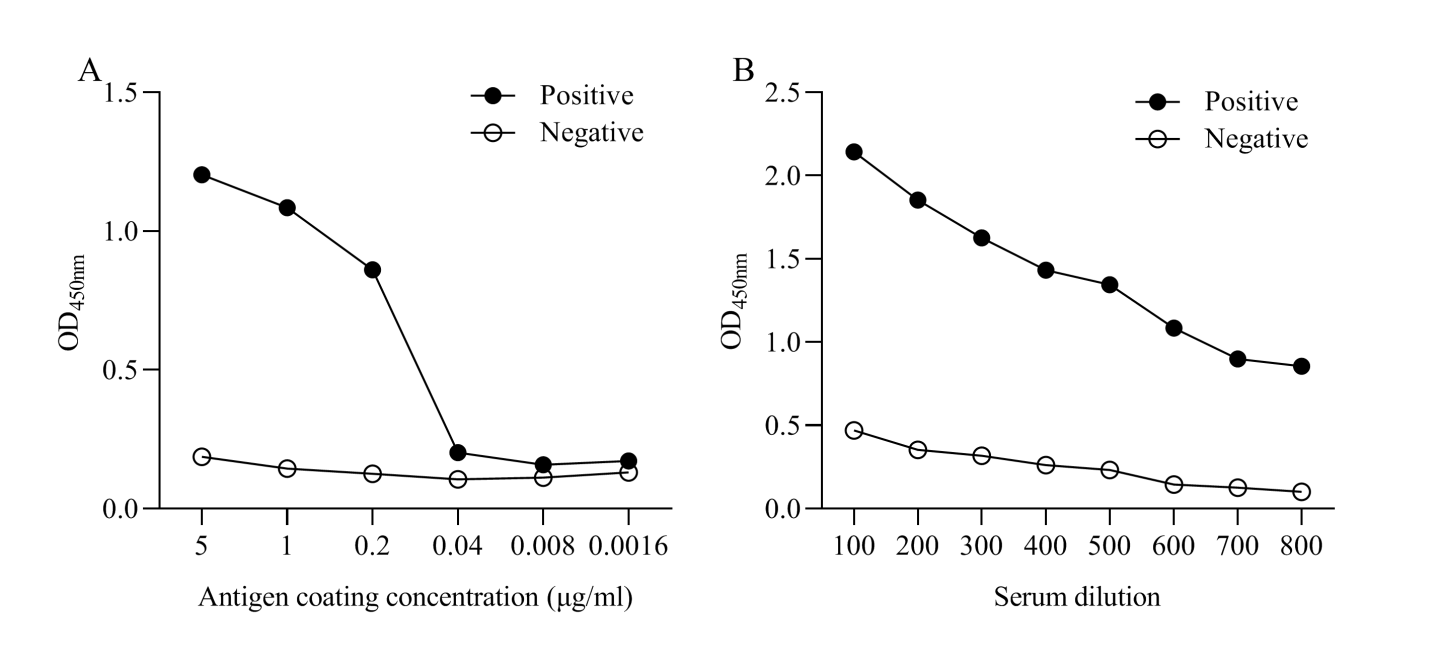


**Supplementary FIGURE S3. Determination of the optimal coating concentration of rLP78 and the optimal dilution tested chicken serum**. The optimal concentrations of the coating antigen and serum dilution were determined through checkerboard titration. Briefly, rLP78 was serially diluted 5-fold from 5 μg/mL to 1.6 ng/mL in sodium carbonate buffer (0.05 mol/L, pH 9.6). Eight dilutions ranging from 1/100 to 1/800 were prepared for positive and negative serum samples. The optimal conditions were expected to show the greatest ratio of OD_450nm_ of positive and negative sera (P/N ratio), with an absorbance of > 1.0 for positive sera and < 0.15 for negative sera. (**A**) Reactions of different concentrations of rLP78 with 1/600 dilution of anti-*M. synoviae* positive and negative serum from chickens. (**B**) Reactions of different dilutions of anti-*M. synoviae* positive and negative serum from chickens with 1 μg/mL of rLP78.

**Supplementary Tables**

**Supplementary TABLE S1. Primers used for site-directed mutagenesis and overlap PCR amplification of lp78 gene.**

| Primer names | Sequence 5’-3’ | Length | Amplicon size (bp) |
| --- | --- | --- | --- |
| lp78-1-F | GGAGATAAAACTGATGGTGGATCTG | 25 | 99 |
| lp78-1-R | GTTATTAATTCTTTTCCATTCTTCAGGAG | 29 |  |
| lp78-2-F | CTCCTGAAGAATGGAAAAGAATTAATAAC | 29 | 126 |
| lp78-2-R | CTTCTCCTTCGCTCCATGGAGCACCAAG | 28 |  |
| lp78-3-F | CTTGGTGCTCCATGGAGCGAAGGAGAAG | 28 | 552 |
| lp78-3-R | ACCCCACAGTTCTTTAACTGCTTC | 24 |  |
| lp78-4-F | GAAGCAGTTAAAGAACTGTGGGGT | 24 | 482 |
| lp78-4-R | GATCTTCCCCAGTGTGATGTTGAAACA | 26 |  |
| lp78-5-F | TGTTTCAACATCACACTGGGGAAGATC | 26 | 611 |
| lp78-5-R | CATTTTTCCATTTTCCAGGAACA | 22 |  |
| lp78-6-F | TGTTCCTGGAAAATGGAAAAATG | 22 | 129 |
| lp78-6-R | GATAGAAGCCATTTTAGGAATAATCTTGTAG | 30 |  |
| lp78-7-F | CTACAAGATTATTCCTAAAATGGCTTCTATC | 30 | 398 |
| lp78-7-R | GTTGTTAGTAGCTCTAACGGTTGATACAA | 29 |  |

**Supplementary TABLE S2.** **Seroprevalence of *M. synoviae* infection in different provinces in China.**

| Provinces | No. of sera tested | No. of positive sera | Positive rates % | No. of Flocks | No. of  positive flocks | Positive rates % |
| --- | --- | --- | --- | --- | --- | --- |
| Shaanxi | 568 | 253 | 44.5 | 9 | 6 | 66.7 |
| Henan | 765 | 446 | 58.3 | 11 | 8 | 72.7 |
| Shanxi | 432 | 294 | 68.1 | 7 | 5 | 71.4 |
| Guangxi | 275 | 206 | 74.9 | 5 | 5 | 100 |
| Total | 2040 | 1199 | 58.8 | 32 | 24 | 75.0 |
